# The use of a minimally invasive integrated endoscopic system to perform hemilaminectomies in chondrodystrophic dogs with thoracolumbar intervertebral disc extrusions

**DOI:** 10.3389/fvets.2024.1296051

**Published:** 2024-04-24

**Authors:** Brittany MacQuiddy, Lisa Bartner, Angela Marolf, Sangeeta Rao, Emily Dupont, Taylor Adams, Eric Monnet

**Affiliations:** ^1^Department of Clinical Sciences, College of Veterinary Medicine and Biomedical Sciences, Colorado State University, Fort Collins, CO, United States; ^2^Department of Veterinary Clinical Sciences, College of Veterinary Medicine, The Ohio State University, Columbus, OH, United States; ^3^Department of Small Animal Clinical Sciences, School of Veterinary Medicine and Biomedical Sciences, Texas A&M University, College Station, TX, United States

**Keywords:** minimally invasive spinal surgery, endoscopic hemilaminectomy, intervertebral disc extrusion, chondrodystrophic, dog

## Abstract

**Introduction:**

The objective was to evaluate the use of a minimally invasive surgical (MIS) approach to perform hemilaminectomies in chondrodystrophic dogs with thoracolumbar intervertebral disc extrusions (IVDE). Additionally, we aimed to evaluate the degree of soft tissue trauma using the endoscopic procedure compared to the standard open approach.

**Methods:**

Eight client-owned dogs presented to the Colorado State University Veterinary Teaching Hospital with acute onset thoracolumbar IVDE were included in this study. This was a prospective, randomized case-series. Patients were assigned to undergo an endoscopic (group 1; *n* = 4) or a standard open approach (group 2; *n* = 4) for a hemilaminectomy. A post-operative MRI was performed in all cases.

**Results:**

Conversion to an open approach was not necessary for any case in group 1. All cases had adequate spinal cord decompression on post-operative MRI. There was no significant difference in soft tissue changes noted on post-operative MRI between the two groups.

**Discussion:**

The MIS approach to hemilaminectomies in chondrodystrophic dogs with thoracolumbar IVDE can successfully be performed to decompress the neural tissue and appears to lead to similar clinical outcomes in the early postoperative period compared to the standard open approach. Larger studies are needed to determine the potential advantages of the MIS technique compared to the standard open approach in veterinary medicine.

## 1 Introduction

Minimally invasive approaches to spinal surgeries have been well described in human medicine with advancements rapidly increasing in the past two decades. This ever-growing focus began in 1999 with the development of a micro-endoscopic discectomy approach (1). The proposed advantages of a minimally invasive surgical (MIS) approach to the spine over an open approach include reduced soft tissue trauma, decreased blood loss, faster surgery times, shorter recovery times, decreased post-operative pain, and lower complication rates (2–5). Different techniques exist for MIS approaches to the spinal cord that utilize either direct visualization of the instruments (via a small skin incision) or indirect visualization (percutaneously).

There have been limited investigations into the utility and feasibility of endoscopic spine surgery in veterinary medicine. The first description was the endoscopic-assisted L7-S1 foraminotomy performed in six healthy large breed dogs (6). Since then, other endoscopic-assisted approaches have been described in both cadavers and live animals (7–11). The use of an entirely endoscopic approach to spinal surgery in dogs has only been described in two previous cadaveric studies (8, 12). Within the thoracolumbar region, approaches for lateral corpectomy (7, 13), pediculectomy (9), foraminotomy (8), foraminotomy with partial corpectomy (10, 11), and mini-hemilaminectomy and discectomy (14) have been reported; only a single report of a hemilaminectomy exists (12). The use of MIS approaches in dogs with naturally occurring disease is limited to a single case report (11) and a retrospective clinical study (14), with another study simulating natural disease using barium-impregnated agarose gel (8).

A recent study was performed using the minimally invasive integrated endoscopic system for hemilaminectomies in large-breed cadaver dogs (12). This was shown to be a reliable approach to access the spinal canal in large breed dogs. However, intervertebral disc extrusions (IVDE) more commonly occur in small, chondrodystrophic breeds (15–17). A previous study evaluated eleven dogs that underwent a microendoscopic mini-hemilaminectomy and discectomy in small breed dogs; however, this was based on retrospective data and not directly compared to any other approaches (14). The MIS technique may be a viable alternative to the standard open approach hemilaminectomy; however, there are no prospective clinical studies that evaluate the use of an endoscopic approach in small-breed dogs with naturally occurring IVDE in the thoracolumbar region with direct comparison to the standard open approach.

Thus, the current study’s purpose was to evaluate the use of an endoscopic approach to perform hemilaminectomies in chondrodystrophic dogs with intervertebral disc extrusions. Additionally, comparisons between the endoscopic technique and the standard open approach were made to evaluate the level of soft tissue trauma and postoperative recovery. We hypothesize that the use of the MIS technique for performing hemilaminectomies will successfully decompress the spinal cord and surrounding nervous tissues. Furthermore, we hypothesize that there will be less soft tissue trauma and a faster post-operative recovery when comparing the endoscopic approach with the open approach hemilaminectomy.

## 2 Materials and methods

### 2.1 Case selection criteria and data recorded

In this prospective, short case series, eight client-owned dogs were enrolled based on the inclusion criteria described below. Patients were randomized to receive either the endoscopic approach or the standard open approach using excel. Randomization took place prior to the start of the study. All cases presented to the Colorado State University Veterinary Teaching Hospital (CSU-VTH) Urgent Care service between September 2020 and September 2021. Informed consent was obtained from all owners prior to enrollment in this study. Dogs were enrolled in the study if they met the following inclusion criteria: chondrodystrophic breed, body weight of <15 kg, acute paraplegia (<2 weeks) with neurolocalization to T3-L3 myelopathy, intact nociception, or absent nociception for <48 h at the time of surgery, single site intervertebral disc extrusion causing focal spinal cord compression, and otherwise healthy. Dogs were excluded if there were concurrent comorbidities or systemic disorders, previous or concurrent spinal cord conditions, chronic myelopathies (>2 weeks), and lacking nociception for >48 h. Clinical presentation, breed, age, sex, reproductive status, body weight, and vital parameters of each dog were recorded.

On clinical presentation, a neurologic examination was performed by a neurology intern and faculty neurologist (LB, BM) to determine eligibility for pre-enrollment. The duration of severe neurologic dysfunction (i.e., paraplegia) at the time of presentation was categorized as <24 h, 24–48 h, or > 48 h from onset. Dogs were assigned a neurologic grade at presentation based on the criteria represented in Table 1 (18, 19). If present, spinal shock was recorded. Spinal shock was defined as transient loss of muscle tone and segmental spinal reflexes caudal to the site of spinal cord injury (20). By virtue of our inclusion criteria, only grade 4 and 5 dogs were considered for enrollment.

**Table 1:** Neurologic grading system (0–5).

**Grade 0**	Normal
**Grade 1**	Pain only, no neurological deficits
**Grade 2**	Paraparesis-ambulatory
**Grade 3**	Paraparesis- non-ambulatory
**Grade 4**	Paraplegia with nociception
**Grade 5**	Paraplegia without nociception

**Figure 1:**
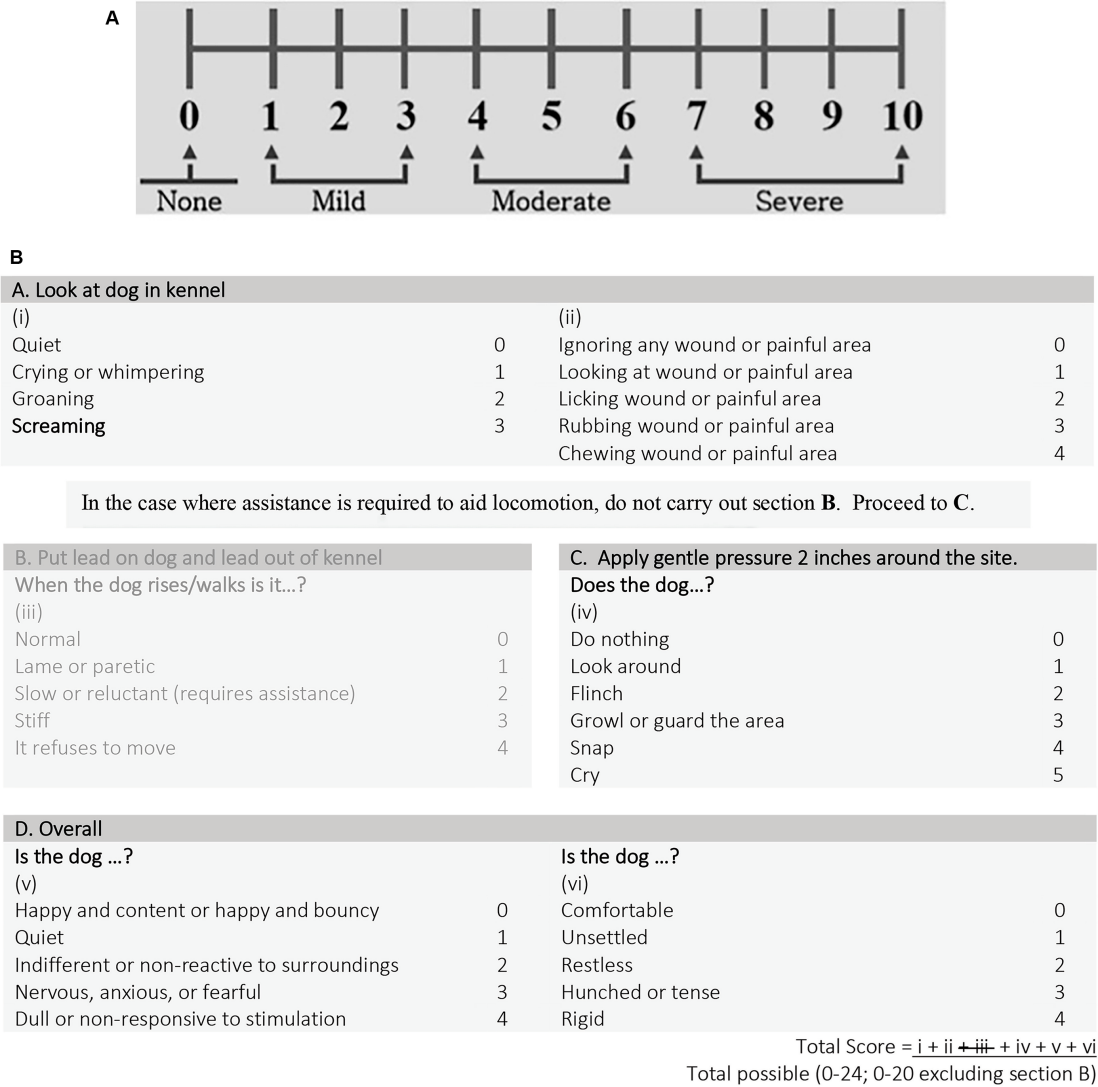
**(A)** Numeric pain scale (NPS) and **(B)** short form Glasgow composite measure pain scale (CMPS-SF).

Once the patient was approved for pre-enrollment, a complete blood count, chemistry (including creatine kinase), pre-anesthetic pain assessment (t0), and thoracolumbar magnetic resonance imaging (MRI) were performed. Thoracic radiographs were performed at the clinician’s discretion. Dogs were excluded if there was any significant abnormality on baseline bloodwork. Pain assessments seen in Figure 1 were made using the Numeric Pain Score (NPS, 0 [none] to 10 [most severe]) and the short form of the Glasgow Composite Pain Scale (CMPS-SF) as previously described (21). Since only non-ambulatory dogs were considered for enrollment, section B in the CMPS-SF was eliminated, allowing a total score between 0 (no pain) and 20 (most severe pain). A single blinded pain observer (ED or TA) was randomly assigned to each dog and was the pain observer for the entirety of the patients’ hospitalization when possible. Both pain observers were small animal rotating interns during the study period. They did not receive any formal training on pain assessment prior to the start of the clinical trial. Final inclusion into the study was based on thoracolumbar MRI confirmation of a single site of IVDE and observations during surgery. Only non-dispersed IVDE were included. Eight cases were randomly assigned to undergo an MIS (group 1; *n* = 4) or a standard open (group 2; *n* = 4) approach for a hemilaminectomy. All dogs underwent a post-operative MRI to ensure adequate spinal cord decompression.

Five of the eight patients were not administered pre-medications prior to anesthesia because these patients had a previously placed intravenous catheter that allowed for rapid induction without pre-medication. Two patients were pre-medicated with hydromorphone (0.05–0.1 mg/kg IV/IM) and atropine (0.02 mg/kg IV/IM). One patient was pre-medicated with butorphanol (0.2 mg/kg IM). Induction agents used were either propofol alone, propofol in combination with fentanyl/ketamine, combination of fentanyl/ketamine/alfaxalone, or combination fentanyl/ketamine/midazolam at variable dosages. All patients were maintained under anesthesia with isoflurane in 100% oxygen adjusted based on anesthetic depth. During surgery, patients were administered fentanyl and ketamine constant rate infusions (CRI), titrated to maintain adequate analgesia. Cefazolin (22 mg/kg IV) was administered just prior to surgery and then every 90 min intra-operatively. Due to scheduling and equipment availability, six patients underwent two separate anesthesia events for neuroimaging and surgery.

Duration of anesthesia (including pre-operative MRI, surgery, and post-operative MRI), duration of surgery, incision length, intra-operative and anesthetic complications, and subjective measurements including intra-operative hemorrhage (1–3 scale, 1-mild to 3-severe), intra-operative visibility of the spinal cord, nerve root, and extruded disc (1–3 scale, 1-worst to 3-best), degree of decompression (1–3 scale, 1- worst to 3-best) were recorded for each procedure. Postoperative pain assessment was performed by one of two blinded observers every hour for the first eight hours following surgery with t1 (time point 1) being one-hour post-extubation, then every 12 h for the duration of hospitalization. Bandages were placed over all incisions for the duration of the patients’ hospitalization to prevent pain observer bias. Daily neurologic examinations with neurologic grade (0–5; Table 1), daily post-operative serial pain scores (NPS and CMPS-SF), and daily post-operative creatine kinase (CK) were recorded for each patient for four days following surgery.

### 2.2 Magnetic resonance imaging and measurements

Pre- and post-operative MRI was performed using a 1.5 T MRI unit (General Electric Signa LX 1.5 Tesla MR HiSpeed Plus System, GE Medical Systems, Milwaukee, WI). Examinations included the following pulse sequences in the transverse and sagittal planes with slice thickness of 2-mm: T1-weighted (T1W), T2-weighted (T2W), proton density (PD), and T2-weighted gradient recalled echo (T2*). T1W post-contrast sequences were fat saturated and completed in transverse, sagittal, and dorsal planes. Post-operative MRI was performed within 48 h of surgery. The imaging parameters including relaxation time (TR), time to echo (TE), and flip angle are summarized in Table 2.

**Table 2:** Magnetic resonance imaging parameters.

	TR (msec)	TE (msec)	Flip angle
T2W	2,265–5,414	100.1–106.7	90̊
T2*	350	7.7–8.8	20̊
PD	2,500	9.9–10.5	130̊
T1W	488–654	10.1–16.7	90̊
T1W FS + C	416–673	10–15.2	90̊

TR = relaxation time, TE = time to echo, T2W = T2-weighted, T2* = T2-weighted gradient recalled echo, PD = proton density, T1W = T1-weighted, T1W FS + C = T1-weighted fat saturated post-contrast.

**Figure 2:**
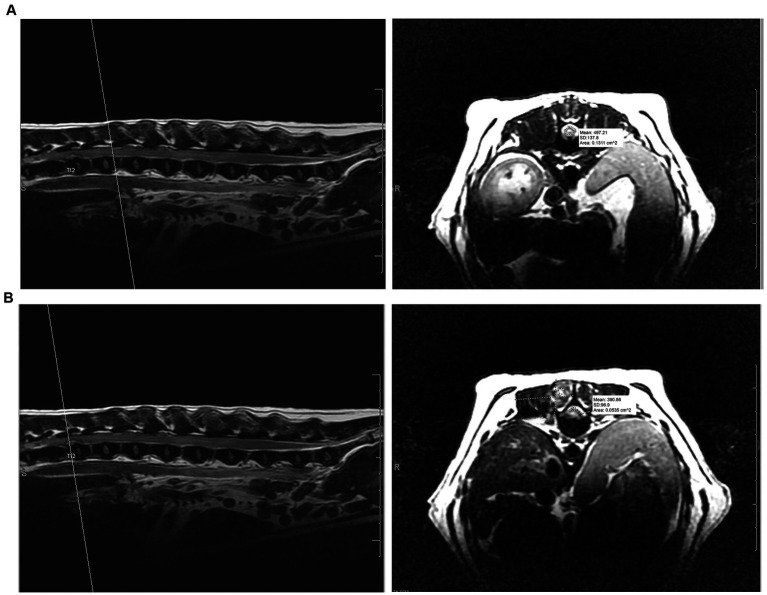
**(A)** Pre-operative measurement of cross-sectional area at unaffected site versus **(B)** the pre-operative measurement of cross-sectional area at the area of maximum spinal cord compression to determine degree of spinal cord compression on T2-weighted transverse and sagittal MRI sequences.

A board-certified radiologist (AM) blinded to the type of procedure performed, reviewed all MR imaging studies and performed all measurements for consistency. Information regarding the site and laterality of IVDE, degree of spinal cord compression, and muscle signal change and dimensions at the affected site were recorded from the pre-operative MRI. The calculation for the degree of spinal cord compression using the cross-sectional area (CSA) is as follows: ([CSA of unaffected spinal cord − CSA of spinal cord under maximal compression]/CSA of unaffected spinal cord) × 100 (22). The CSA was evaluated on T2W transverse images by tracing the outline of the spinal cord at both an unaffected site and the site of maximal compression. Post-procedure the degree of spinal cord compression, size of the laminectomy window in millimeters (length and height), and muscle intensity and dimensions around the hemilaminectomy site were recorded from the post-operative MRI. Examples of pre-operative MRI measurements of cross-sectional area to determine the degree of spinal cord compression can be seen in Figure 2. The hemilaminectomy window’s size was determined by measuring the height from the dorsal margin of the laminectomy in the vertebral canal to the ventral margin at the level of the dorsal annulus of the intervertebral disc (IVD) and the site’s length from the cranial margin to the caudal margin of the laminectomy. These measurements were performed on the PD sequences to highlight bone more readily with the transverse and sagittal sequences used in tandem with a scout line to ensure the cranial and caudal extents of the length of the hemilaminectomy. The degree of muscle hyperintensity and size change was evaluated for both approaches by comparing the dimensions in mm on T2-weighted images (width, height, and depth) and the contrast enhancement on T1-weighted FSE and TI-weighted Fat saturated post-contrast images from the pre- and post-surgical studies. When compared to pre-procedure (presumed normal) muscle tissue, the degree of contrast enhancement after the surgical procedure was scored subjectively as follows: up to 25% greater was considered mild, 25–50% greater was moderate, and > 50% was severe. Additionally, to measure the degree of post-operative muscle intensity change more objectively for both approaches, a region of interest (ROI) evaluating paraspinal muscle intensity at the site of intervertebral disc extrusion was selected on T2W, T1W pre- and post-contrast sequences, measuring 5 mm in diameter. The intensity changes based on ROI on the sequences were compared pre- and post-procedure. We attempted to subjectively determine the degree of post-operative hemorrhage based on MRI.

### 2.3 Surgical procedures

**Figure 3:**
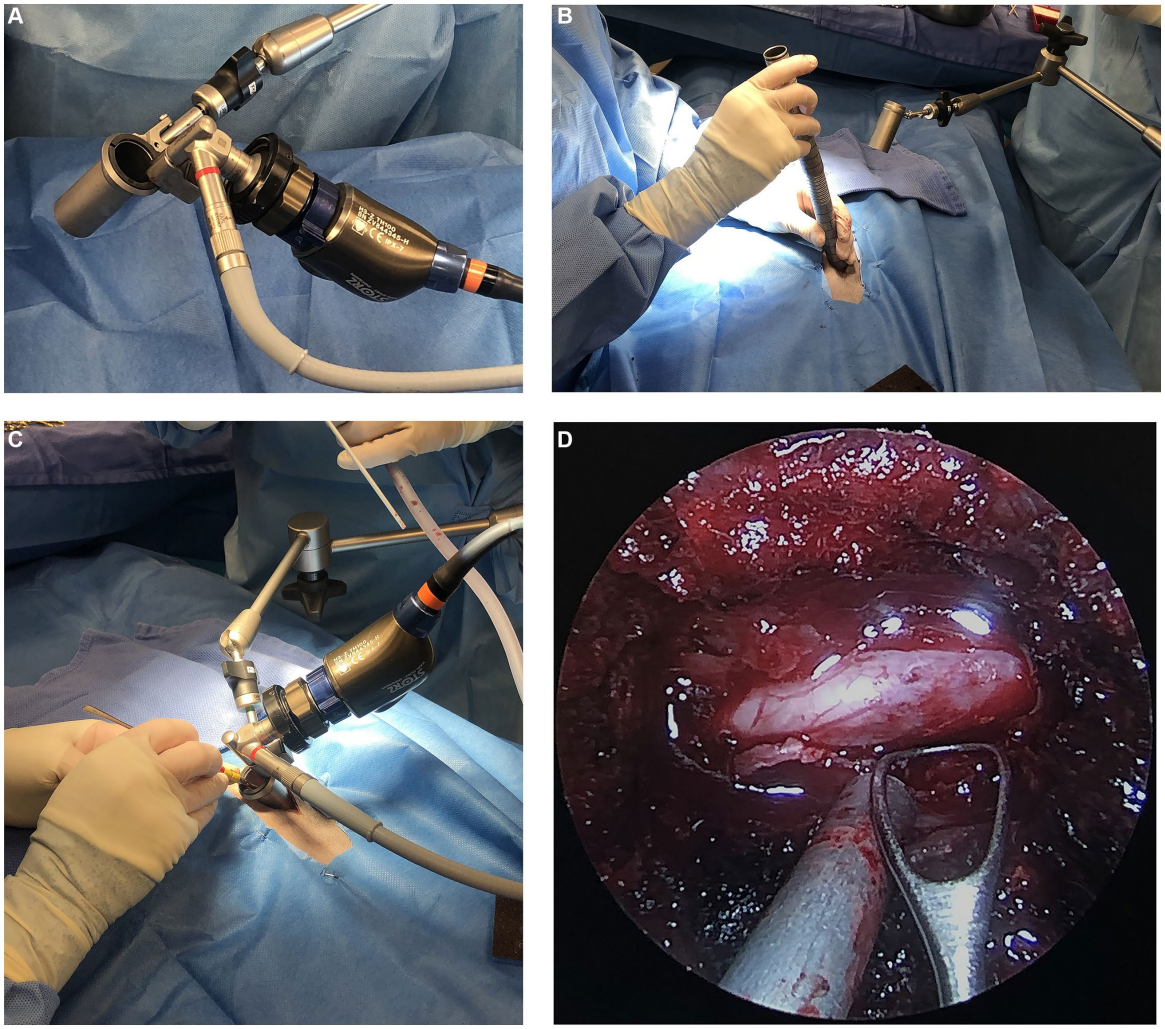
**(A)** Image of the EasyGo! Endoscopic surgical system **(B)** intra-operative image of the use of serial dilator sleeves **(C)** intra-operative image displaying the position of the endoscopic equipment including the trocar, articulating arm, endoscope, and light source **(D)** endoscopic view of the surgical site showing the exposed spinal cord.

All surgical procedures were performed by either a single board-certified neurologist (LB), a single board-certified surgeon (EM), or both. The MIS technique for hemilaminectomies was performed with an integrated endoscopic surgical system (EasyGO! 2nd generation; Karl Storz, Goleta, California) which can be seen in Figure 3A. One of the surgeons (EM) was involved in a prior cadaver study using the EasyGO! system and possesses extensive experience in endoscopy (12). Prior to initiation of the clinical trial, the other surgeon (LB), used the EasyGo! system on several cadavers to gain experience with the instrumentation. Patients undergoing the MIS procedure were placed in sternal recumbency with the table slightly angled away from the surgeon. The site was localized using fluoroscopy. A 25-guage needle was inserted into the articular processes at the level of the affected IVD space. A paramedian incision was made using monopolar electrocautery on the side of the IVDE. The length of the incision was determined by the trocar size in order for the skin to sit securely around the trocar. For the first MIS procedure, the subcutaneous tissues and epaxial muscles were elevated using monopolar cautery, periosteal elevators, and without the use of the trocars. This was done for the surgeons’ benefit, which allowed them to become more comfortable with the instrumentation. The remainder of the MIS procedures were performed entirely through the endoscopic system. Once the articular processes of interest were isolated, a 0.035-inch Steinman pin was inserted into the articular processes. The first dilation sleeve was fitted over the pin and pushed toward the base until it was oriented 45-degrees laterally from the spinous process and in firm contact with the articular processes. Maintaining this angle throughout the serial placement of incrementally increasing sizes of dilator retractors was critical in allowing the final trocar to rest in parallel with the lamina. The trocar size (19 mm vs. 23 mm) was selected for the maximum diameter that fit between the articular processes while maintaining contact with the lamina. The remainder of the approach to the vertebral canal was performed as described (12). Figures 3A–D include images of the endoscopic equipment, use of the serial dilator sleeves, and endoscopic view of the spinal cord. Once in the vertebral canal, the extruded disc material was visualized and removed with fine instruments. The endoscopic trocar could be re-directed cranially or caudally by minute adjustments in a fanning motion, maintaining the point of contact between trocar and lamina as a fulcrum, figuratively. After adequate spinal cord decompression was achieved, a porcine small intestinal submucosa graft (Vetrix BioSIS T; Cook Biotech, Wisconsin) was placed over the laminectomy window and a standard 3-layer closure was performed. No topical or infiltrative analgesic medications were used to avoid obscuring interpretation of muscle changes on MRI or post-operative pain assessments.

Patients for the standard open approach hemilaminectomies were placed in sternal recumbency. Fluoroscopy was used to confirm the location of the surgical site. An incision slightly lateral to the spinous processes was made using monopolar electrocautery. The length of the incision was created to allow for adequate exposure of the accessory processes at the site of intervertebral disc herniation by extending one site cranially and caudally to the site of herniation. The subcutaneous tissues were dissected to expose the lumbodorsal fascia. Monopolar electrocautery was used to make scalloped incisions around the spinous processes into the lumbodorsal fascia, taking care to ensure enough fascia remains for proper closure and continued to elevate the multifidus musculature off the spinous, articular and accessory processes. Through a dorsolateral approach The laminectomy window included drilling through the articular processes at the affected site with the ventral border abutting but not including the accessory process. Monopolar and bipolar electrocautery were used for muscle dissection, removal of ligamentous attachments, and hemostasis. Gelpi retractors were used to maintain visualization of the surgical site. The majority of the hemilaminectomy was performed as previously described (19, 23, 24).

If a patient undergoing an MIS hemilaminectomy had to be converted to an open approach, a postoperative MRI would not be performed. If the post-operative MRI of the MIS hemilaminectomy did not show adequate decompression at the surgical site, then the patient was taken back in for surgery and converted to an open approach. At the time of surgery, if the affected site was determined to be a chronic disc herniation, then the patient was excluded from the study. Fenestration of the intervertebral disc was not performed in either of the groups at any site.

### 2.4 Post-operative care

All patients recovered in the Intermediate Care Unit of the CSU-VTH. The incision sites were iced upon recovery and then every six hours for the first 24 h following surgery. The time of extubation was noted as a reference for the pain observers. The CMPS-SF and NPS were performed every 60 min for the first eight hours and then every 12 h until discharge (i.e., 1, 2, 3, 4, 5, 6, 7, 8, 12, 24, 36, 48, 60, 72, and 84 h post-extubation). Following recovery, all patients were maintained with maintenance IV fluid therapy, fentanyl CRI 2–5 mcg/kg/h, gabapentin 10 mg/kg orally every 8 h, prazosin 1 mg/15 kg orally every 8 h, and prednisone 0.5 mg/kg orally every 12 h. Some dogs were administered trazodone 3–5 mg/kg orally every 8 h for anxiety in hospital and for at-home use. If a patient had a pain score ≥ 4 for NPS or > 12 for CMPS-SF, rescue analgesics would be provided which could include the addition of intravenous methadone 1–3 mg/kg, ketamine CRI 2–4 mcg/kg/min, lidocaine CRI 10–20 mcg/kg/min, or oral amantadine 3 mg/kg every 12 h. Urinary catheters were placed prior to surgery and maintained for 12–24 h following surgery. Intravenous fluid therapy and fentanyl CRIs were discontinued within 24–48 h of surgery once the patient was comfortable on oral medications. Rehabilitation exercises were not initiated in any patient during hospitalization. Patients were all discharged on the fourth day following surgery with instructions for restricted activity, two-week tapering course of prednisone, gabapentin, and prazosin, with or without trazodone at the previously described dosages. Owners were given quality of life (QOL) questionnaires to be filled out daily for the first seven days after discharge (see Supplementary material).

### 2.5 Follow-up

All patients were represented to the CSU Neurology Clinical Trials service for a neurologic examination, suture removal, and pain assessment two weeks after surgery. Quality of life forms were collected at this follow-up. This consisted of ten score rating questions and nine yes or no questions (see Supplementary material). A neurologic grade was recorded. The incision was briefly covered for the final pain assessment. At the two-week recheck, the patients had completed their enrollment in the study. Recommendations for starting rehabilitation therapy were made during this visit. Information regarding long-term follow-up for any patient until the point of writing will be included.

### 2.6 Statistical analysis

Due to small numbers in each group, the continuous data was described using medians and ranges and analyzed using a non-parametric approach of Wilcoxon 2-sample test. The categorical data was described using frequencies and compared using a Fisher’s exact test. A *p*-value of 0.05 was used to determine statistical significance. The QOL questionnaire scores were described using medians and analyzed using non-parametric Wilcoxon 2-sample test. The categorical data were analyzed using a Chi-square test. If any of the categories were < 5, then the data were analyzed using Fisher’s exact test. A *p*-value of 0.05 was used to determine statistical significance. SAS v9.4 (SAS Institute Inc., Cary, NC) was used for all statistical analyses. A power analysis was performed prior to the start of the study. To be able to find a difference between the proportion of paralyzed dogs with nociception and those without, a total of 52 dogs would be required to enroll in the study (26 in each group) to achieve a power of 80% and a statistical confidence of 95%.

## 3 Results

### 3.1 Case population

Nine dogs were initially enrolled in the study. One patient enrolled in group 2 was excluded at the time of surgery due to a chronic intervertebral disc protrusion at the affected IVD space. Of the eight dogs that completed enrollment, there were five females (2 intact, 3 spayed) and three males (1 intact, 2 castrated) enrolled in the study. There were two shih tzus, two mixed breeds, and one of each of the following breed: toy poodle, cocker spaniel, French bulldog, and miniature dachshund. The median age at presentation for group 1 and group 2 was 3.3 years (range: 2.7–5.7 years) and 4.9 years (range: 3–10 years), respectively. The median weight for group 1 and group 2 was 5.9 kg (range: 3.1–11.8 kg) and 6.9 kg (range: 5.5–10.4 kg), respectively. Patient data is summarized in Table 3. The duration of severe neurologic signs was present for <24 h in five dogs, 24–48 h in one dog, and > 48 h in two dogs. The results of the hematology and serum biochemistry analyses showed no clinically relevant abnormalities or warranted exclusion from the study. Thoracic radiographs were performed in three patients and revealed a normal thoracic study. All dogs had a single site IVDE within the T3-L3 spinal cord segments except for one. Patient 3 had an IVDE at L4-L5 but based on neurologic examination, localized to T3-L3 myelopathy, and was therefore included in the study. At the time of presentation, six dogs had a neurologic grade of 4 (4 in group 1; 2 in group 2) and two dogs had a neurologic grade of 5 (group 2). Three dogs (1 in group 1; 2 in group 2) had spinal shock at presentation.

**Table 3:** Case information for the 8 dogs in the study.

Patient#	Age (years)	Sex	Breed	Weight (kg)	Duration of severe signs	IVDE site	Laterality	MIS vs standard
**1**	3.6	FS	Toy poodle	5.7	<24 h	T12T13	R	MIS
**2**	3	FS	Cocker spaniel	11.1	24-48 h	T13L1	L	MIS
**3**	5.3	MC	French bulldog	9.7	>48 h	L4L5	L	Standard
**4**	10	MC	Mixed breed	5.9	<24 h	T13L1	L	Standard
**5**	4.5	FS	Shih tzu	6.3	<24 h	T13L1	L	Standard
**6**	2.7	FI	Miniature Dachshund	3.4	<24 h	L1L2	R	MIS
**7**	5.7	FI	Mixed breed	5.8	>48 h	T13L1	R	MIS
**8**	3	MI	Shih Tzu	5.5	<24 h	L2L3	R	Standard

FS = female spayed, FI = female intact, MC = male castrated, MI = male intact, IVDE = intervertebral disc extrusion, R = right, L = left, MIS = minimally invasive surgery. #Patient ID number.

### 3.2 Intra-operative measurements and complications

**Figure 4:**
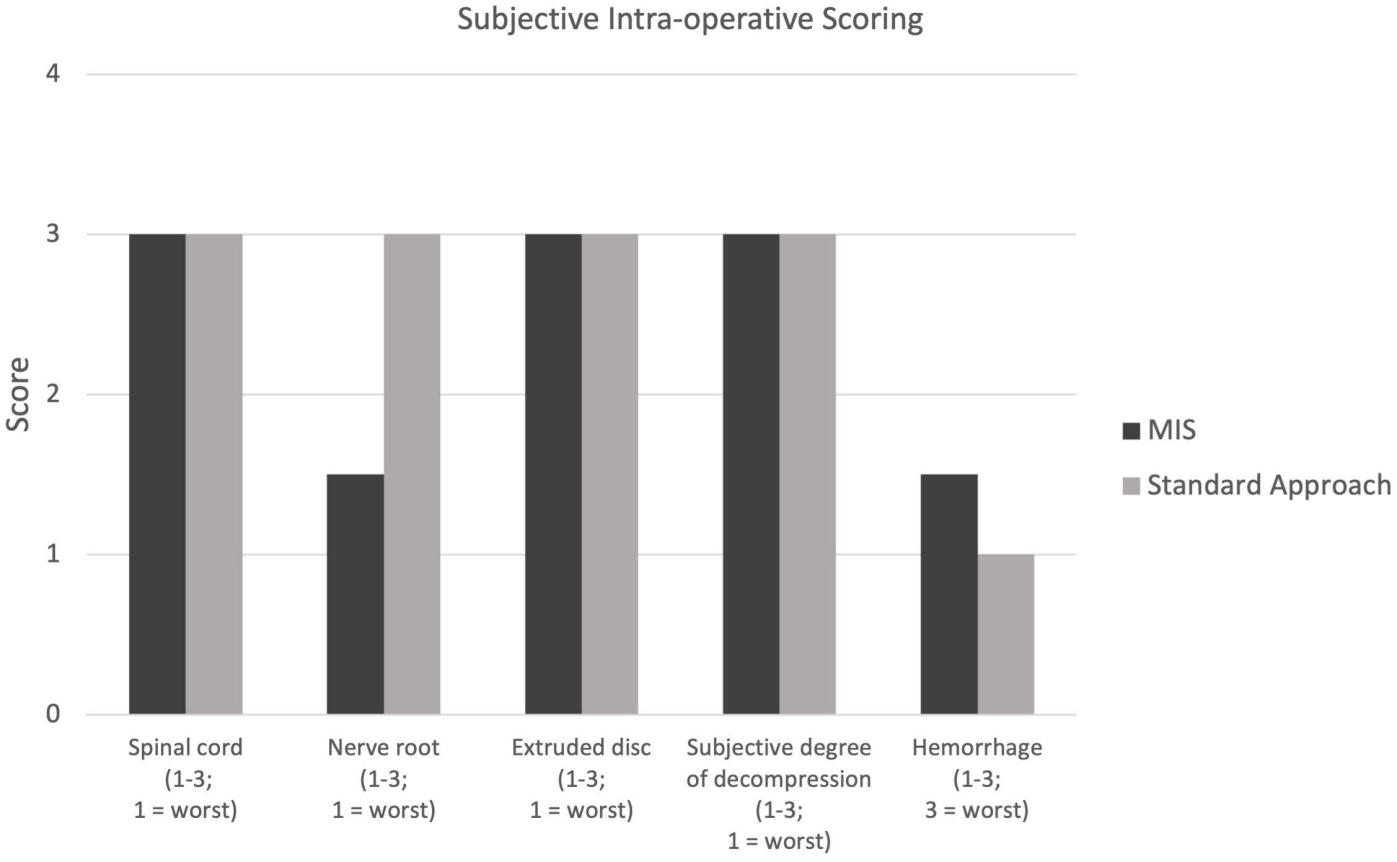
Intra-operative scoring for visualization of the spinal cord, nerve root, and extruded disc (1–3; 1 = worst); scoring for subjective degree of spinal cord decompression (1–3; 1 = worst); scoring of intra-operative hemorrhage (1–3, 3 = worst).

Conversion to an open approach was not required for any dogs receiving the MIS procedure. The median duration of anesthesia for group 1 was 450 min (range: 353–750 min) and for group 2 was 372.5 min (range: 250–770 min). Surgery time was significantly longer (*p*-value = 0.04) in group 1 at 132 min (range 110–137 min) than in group 2 (83.5 min; range 64–110 min) The median incision length was 3.8 cm (range: 3–4.2 cm) for group 1 and was significantly shorter than the 9.3 cm (range: 5.3–10.8 cm) for group 2 (*p*-value = 0.03). The subjective degree of decompression, scored on a 1–3 scale (3 = best) was 2.5 in one case (group 1) and 3 in all remaining cases (3 in group 1; 4 in group 2). All subjective scores including for hemorrhage and visibility of the extruded disc material, spinal cord, and nerve root can be seen in Figure 4. There were no intra-operative complications encountered in either group. The trocar diameter size used for the MIS procedure was 23 mm in two dogs (Dog number 1 and 2) and 19 mm in two dogs (Dog number 6 and 7).

### 3.3 Pre- and post-operative MRI measurements

**Figure 5:**
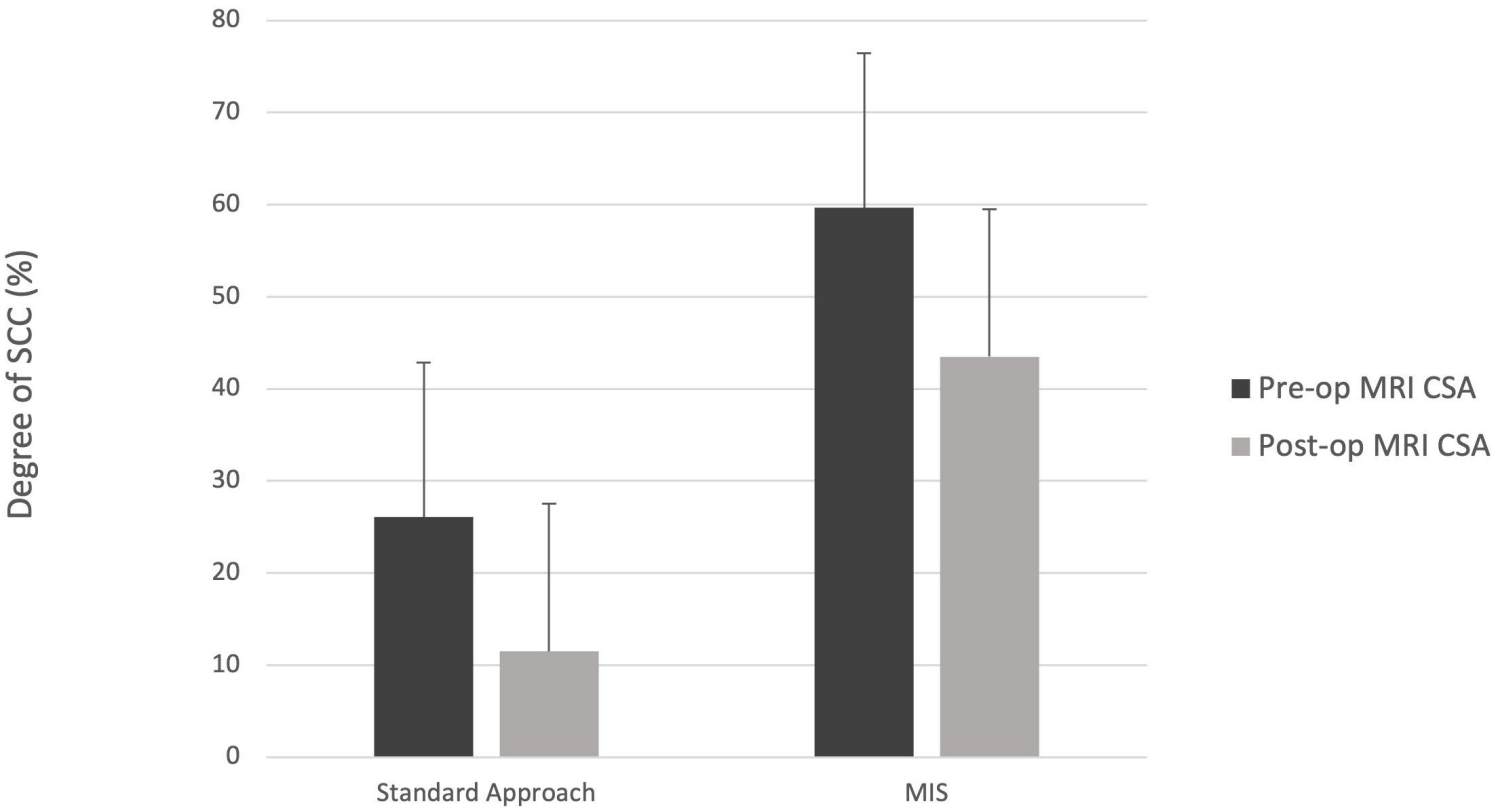
Median degree of spinal cord compression (SCC) for the standard and MIS approach both pre- and post-operatively. CSA = cross-sectional area.

The degree of spinal cord compression measured as cross-sectional area post-operatively was not significantly different when comparing groups 1 and 2 (Figure 5). The degree of spinal cord compression in group 1 was significantly less post-operatively compared to pre-operative (43.5%, range: 27.6–52.6, and 59.7%, range: 53.5–60.1%, respectively). While there was reduction in compression of the spinal cord in group 2, there was no statistically significant difference in the degree of spinal cord compression post-operatively compared to pre-operatively (11.5%, range: 6.8–65.2, and 26.1%, range: 10.8–81.3%, respectively). The median laminectomy height for group 1 and group 2 was 7.6 mm (range: 6.3–8.8 mm) and 7 mm (range: 6–9.9 mm), respectively. The median laminectomy length for group 1 was 19.5 mm (range: 15.9–19.9 mm) and for group 2 was 24.6 mm (range: 16.6–27.6 mm). The laminectomy window size was not statistically different between the two groups.

Following surgery, the depth of soft tissue change observed on T2W images was significantly higher for group 1 and group 2 (*p*-value = 0.03 and *p*-value = 0.03, respectively) compared to pre-operative. However, there were no significant differences in the width and height on pre- and post-operative imaging for either group. Further, there was no significant difference in these dimensions when group 1 was compared to group 2. The degree of contrast enhancement post-operatively was considered mild in one dog (group 2), moderate in one dog (group 1), and severe in six dogs (3 in group 1; 3 in group 2). The ROI obtained on T2W and T1-W + C images for group 2 were significantly higher post-operatively (*p*-value = 0.0304 for both T2W and T1W + C) compared to pre-operatively. This was not found to be significant for group 1. We attempted to determine the amount of post-operative hemorrhage on MR imaging; however, due to the disruption of the normal tissues during surgery, it was difficult to differentiate hemorrhage from gas.

### 3.4 Post-operative complications and clinical outcome

**Figure 6:**
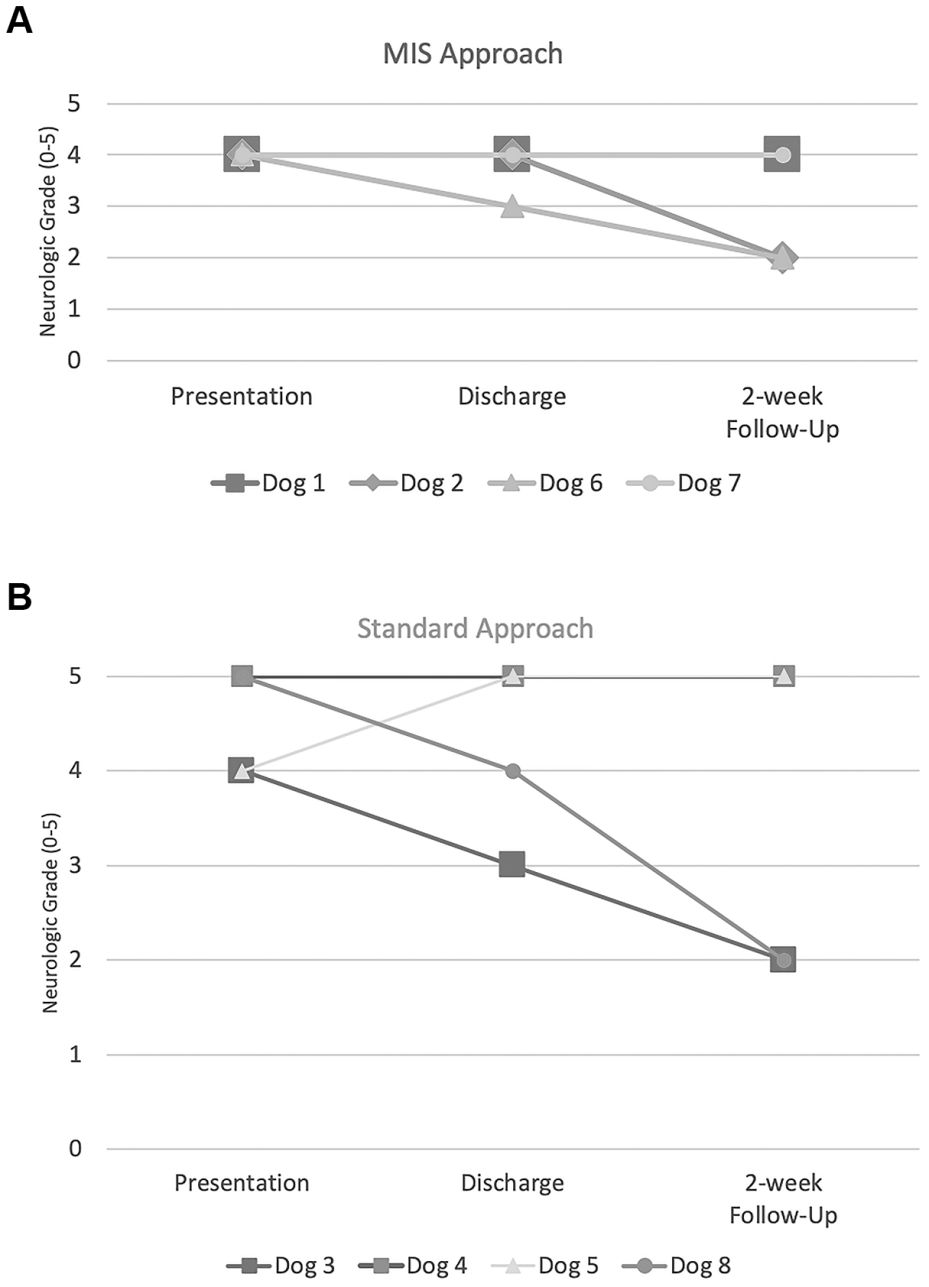
Neurologic grades (0–5) at presentation, discharge, and two-week follow-up for **(A)** MIS approach and **(B)** standard approach.

There were no post-operative complications observed in any of the patients. All dogs in group 1 were a neurological grade 4 on presentation with one dog improving to grade 3 at discharge (Dog number 6). At the two-week recheck, the neurological grades of two dogs improved from discharge to grade 2 (Dog number 2 and 6) and remained unchanged in two dogs with a neurologic grade of 4. Dogs number 1 and 7 improved from a grade 4 at the two-week recheck to grade 2 and grade 3, respectively, at the last follow-up (Dog number 1: 35 days and Dog number 7: 27 days post-operatively). In group 2, two dogs were grade 5 and two dogs were grade 4 on presentation. At the time of discharge, the neurological grade improved in two dogs (Dog number 3 and 8), remained unchanged in one dog (Dog number 4), and worsened in one dog (Dog number 5). At the two-week recheck, the neurological grade improved to grade 2 in two dogs (Dog number 3 and 8) and remained at grade 5 in two dogs (Dog number 4 and 5) from discharge (Figure 6). Dog number 4 developed spinal walking which was noted at subsequent visits. Dog number 5 was still a neurologic grade of 5 at last follow-up 49 days post-operatively.

**Figure 7:**
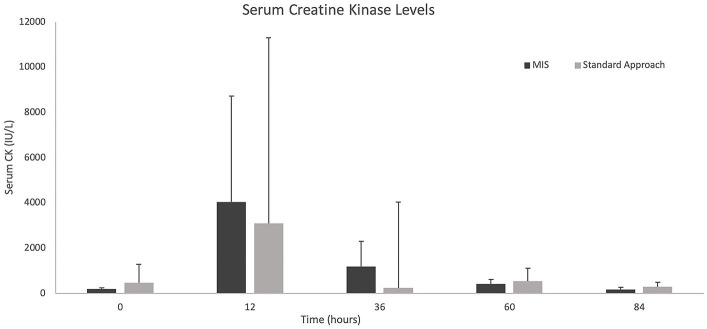
Median +/− standard deviation serum creatine kinase levels at 0, 12-, 36-, 60-, and 84-h time points for the standard and MIS approach.

**Figure 8:**
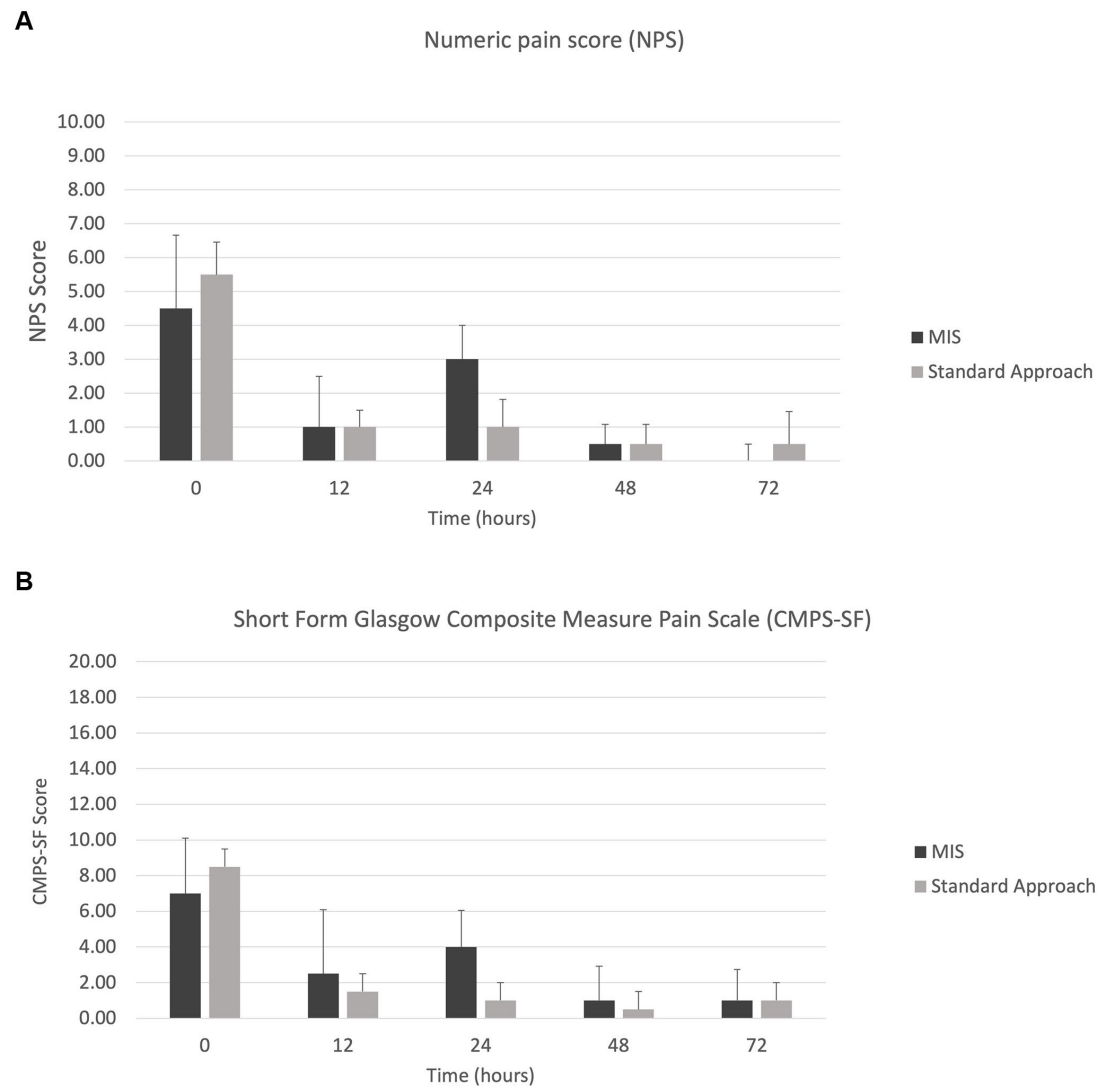
Median +/− standard deviation pain scores for the MIS and standard approach at time points 0, 12-, 24-, 48-, and 72-h for **(A)** numeric pain scale (NPS) and **(B)** short form Glasgow composite measure pain scale (CMPS-SF).

The results of the serum CK values and pain scores (NPS and CMPS-SF) can be visualized in Figures 7, 8. With only four dogs in each group, and wide overlap in CK values, no significant differences were identified. However, in general animals having longer surgery times had higher serum CK levels.

### 3.5 Quality of life questionnaire

All questionnaires were completed for all time points, by all owners. Group 1 was more likely to have difficulty sleeping through the night and to chew or scratch until their skin was irritated compared to group 2. Group 1 was given a median score of 3 compared to a median score of 5 for group 2 when asked if their pet was sleeping through the night (the lower score indicating less likely to sleep through the night). The median score for both groups 1 and 2, when asked if their pet was chewing/scratching themselves and causing irritation, was a score of 2 and score of 1, respectively (the higher score indicating more scratching behavior noted). However, there was no statistical difference between the groups when asked how they think their pet would rate their quality of life.

## 4 Discussion

Minimally invasive approaches for decompressive spinal surgeries have been well documented in human medicine for the past two decades (1, 25–27), boasting numerous advantages over standard open approaches including reduction of intra-operative hemorrhage, post-operative pain, and surgical times, hastened post-operative recovery, and fewer post-operative complications (2–5). In veterinary medicine, a completely endoscopic approach to the thoracolumbar vertebral canal in dogs with naturally occurring intervertebral disc herniations has only been described in a single case report and in a retrospective clinical study (11, 14). Results of this study demonstrate that hemilaminectomies can be successfully completed using this MIS technique. Additionally, the vertebral canal is readily accessed to adequately remove extruded disc material and decompress the nervous tissue in naturally occurring canine spinal cord injuries.

The laminectomy window size was not significantly different between group 1 and group 2. In a previous study, a minimally invasive integrated endoscopic system was used to perform hemilaminectomies in large-breed cadaver dogs (12). They found that the mean length of the hemilaminectomy window measured on Computed Tomography (CT) was 13.0 ± 1.5 mm and 15.0 ± 2.1 mm with a mean height of 10.0 ± 1.9 mm and 10.1 ± 0.9 mm for the 19 mm and 23 mm trocar, respectively. It was concluded that disc material within 10 mm from the disc space caudally and 5 mm from the disc space cranially should be accessible when using a 23-mm cannula in a large breed dog. However, due to interference from surrounding soft tissues, they did not adjust the cannula to extend the surgical site. In the current study, the cannula was often repositioned to extend the laminectomy window. This could explain the difference between the longer lengths of the window in the current study compared to the cadaveric study previously mentioned. One of the criteria for inclusion in the current study was that the patient had to have a single herniated disc causing focal spinal cord compression that did not extend beyond the proposed laminectomy window. Specific measurements of the distance of the displacement of the herniated disc material compared to the intervertebral disc site were not taken. Therefore, we are unable to quantify the limitations of the accessibility through the endoscopic system. Future studies should consider evaluating this limitation in chondrodystrophic breeds clinically affected with thoracolumbar disc herniations.

The duration of surgery was significantly longer for group 1 when compared to group 2. As with other minimally invasive surgeries, it requires training and experience to maximize efficiency. Also, an endoscopic system leads to a longer initial set up time which will lengthen anesthesia and surgical time. In this study, when comparing the MIS approach to the standard approach, a procedure for which both surgeons were proficient, these factors contributed to the longer surgical times in the MIS group. Based on previous studies regarding minimally invasive surgery, the learning curve associated with this approach significantly influences surgery times, complication rates, and clinical outcomes (28–33). When evaluating the initial learning curve of minimally invasive spinal surgery in humans, it seems that performing 30–40 procedures is necessary in order for the surgeon to become proficient at minimizing surgical time and complications (28). There is one study assessing laparoscopic ovariectomy in dogs that required approximately 80 procedures to obtain proficiency and reduce complication rates (34). The surgeon’s in the current study felt that even after 3–4 laminectomies performed in cadavers, their knowledge and capability had greatly improved. In one study of outcomes in 297 nociception negative dogs treated surgically, there was a negative correlation with the time of general anesthesia and regaining ambulatory status (29). This suggests that every effort should be made to limit time under anesthesia in severe spinal cord injury. Due to the added instrumentation of the MIS procedure, the perceived benefits will need to be weighed against patient factors, including the ability to tolerate longer anesthesia. The authors anticipate that with time and experience, a level of proficiency can be attained to mitigate these differences.

The incision length was significantly smaller in group 1 compared to group 2. To ensure proper visualization during a standard open approach to a hemilaminectomy, extensive soft tissue disruption is needed to expose the bony structures of the spine. This helps to identify landmarks of the laminectomy window so that an adequate approach to the spinal cord can be made to allow for maximum visibility and removal of herniated disc material. In the minimally invasive approach, the use of magnification improves visibility; therefore, a larger surgical site exposure and soft tissue disruption is no longer necessary. The popularity of MIS procedures has grown in human medicine, partially attributed to the smaller scar formed. While our veterinary patients do not perceive the smaller incisions, in one study evaluating owners’ perception of outcome, 37% were found to be influenced by visual appearance (35).

Visibility as it relates to the surgeon’s perceived degree of decompression, inspection of the nervous tissue, and any interference from hemorrhage was not hindered through the endoscopic procedure and was objectively as good as a standard open approach. Any amount of hemorrhage during the MIS approach was intensified due to the magnification; however, this allowed for better visibility of the location of the bleeding which lead to quicker hemostasis. All patients, regardless of the type of procedure, had adequate decompression of nervous tissue without intra- or post-operative complications supporting the application of MIS for routine decompression. The nerve root was not visible in two patients in group 1 (Dog number 2 and 6). In these two cases, there was no involvement or compression of the nerve root appreciated on pre-operative MR imaging that would necessitate extending the laminectomy window. In dogs with disc material surrounding the spinal nerves and nerve roots, the MIS trocar could be finely adjusted and fanned in the direction needed. This was readily achievable when warranted. If more extensive fanning were warranted, occasionally muscle surrounding the trocar retractor would creep into the surgical field. This may preclude the efficient use of this MIS system in disc herniations that resulted in more dispersed extruded material. Nerve root compression is a significant cause for pain and if not adequately decompressed, may require a second surgery depending on patient recovery (36, 37). However, the lack of visibility of the nerve root was not clinically important in these two cases. Larger studies are needed to determine the viability of the MIS approach in dogs with nerve root involvement.

The amount of post-operative compression was not significantly different between group 1 and group 2. This suggests that the decompression achieved through the MIS approach was comparable to that of the standard open approach in this small cohort of dogs. Group 1 displayed a greater degree of spinal cord compression before surgery, with a median cross-sectional area of 59.7% compared to 26.1% in group 2, though this was not statistically significant. One might have predicted that there would be more decompression achieved in the MIS group since this group had more compressive lesions than the standard group. An explanation for the lack of this finding could be a more chronic disc extrusion with an acute component in which the spinal cord remained deformed after the removal of the herniated disc material due to fibrosis surrounding the cord. Alternatively, there is a potential that with more time the spinal cord would have returned to a normal shape. However, this conclusion is difficult to draw given the small number of cases and wide range of cross-sectional areas. Therefore, a larger cohort is needed to further investigate the degree of spinal cord decompression and its significance between these two approaches.

Minimally invasive surgery is toted to be sparing to the musculature and ligamentous attachments along the vertebral column, resulting in less soft tissue trauma (2, 3). However, information quantifying this is lacking. The degree of soft tissue change post-operatively was compared with MRI in this study using ROI. When assessing signal intensity of the muscle on T2-W and T1-W post-contrast images, the ROI for group 2 was significantly higher post-operatively compared to pre-operatively but this difference was not found for group 1. This could support more severe soft tissue injury after the standard open approach than for the MIS approach. However, the extent of signal change within the muscle was no different between groups. Given the need for extensive soft tissue disruption during a standard approach, it would make sense that the amount of soft tissue trauma would appear greater for group 2; however, this was not consistent across all MRI measurements. A confounding factor was the longer surgery time in the MIS group which may have made the soft tissue changes more pronounced and be one explanation for the lack of statistical difference between groups 1 and 2. When evaluating dogs with IVDE in a previous study, it was found that those with muscle hyperintensity were younger than the dogs without muscle hyperintensity and half of the affected dogs had evidence of muscle necrosis and intramuscular inflammation. No other factors were found to contribute to the presence of muscle hyperintensity (38). The varying degree of muscle intensity on pre-operative MRI in the current study, which may have numerous and currently unknown predisposing factors, complicates our understanding of the significance of the changes seen post-operatively. Also, we did not evaluate the effect of age on pre-operative MRI muscle hyperintensity. In most patients, post-operative MRI was not performed immediately following surgery due to time constraints. This may have impacted the degree of soft tissue changes noted on post-operative MRI. MRI sequences described at being the best in evaluating muscle edema include Short Tau Inversion-Recuperation (STIR) and DIXON sequences which are both used to suppress fat (39). While T2-W and T1-W post-contrast sequences can also identify muscle edema, they are considered inferior compared to STIR. Additionally, there may be presence of muscle edema or inflammation on a microscopic level that is not hyperintense on these sequences. T1-weighted imaging seems to be better at identifying muscle atrophy and fatty degeneration. It is important to note that there may be other variables that contribute to muscle hyperintensity on T2-W and STIR images such as chemical shift artifact and/or exercise. In the current study, a STIR-sequence was not performed in order to minimize time under anesthesia as the initial hope was to coordinate a pre-operative MRI, surgery, and post-operative MRI in a single anesthesia which was not possible in most patients. In addition, evaluating soft tissue changes was not the main goal of this study. The importance of minimizing soft tissue trauma is to avoid potential long-term complications such as delay in recovery or negative changes to spinal stabilization due to muscle atrophy and fatty infiltration that follows extensive soft tissue damage. In a study comparing percutaneous versus open pedicle screw fixation, there was more extensive paraspinal muscles damage in the open approach; however, there was no significant difference between approaches on short-term outcome (40). Better methods of interpreting soft tissue trauma are needed to determine how this differs between the two approaches on a larger scale. An attempt was made to grade hemorrhage on post-operative imaging, however the voiding artifact hemorrhage created was indiscriminate from air/gas obscuring any findings.

An additional measurement used to evaluate for post-operative muscle injury was serum CK. When evaluating the duration of surgery and CK values, with exceptions, the overall trend indicated that those with a longer surgical time had higher CK values. Serum CK values have previously been measured post-operatively for a minimally invasive spinal surgical approach which showed an initial spike after surgery with slow trend toward normal (10). There are no comparisons of serum CK between severity of neurologic grade or those patients that did not receive surgery. In one study evaluating CK in cerebrospinal fluid (CSF) of dogs with intervertebral disc herniations, it was noted that a higher CK level in CSF correlated with worse outcomes (41). However, this only accounted for a single sample taken at the time of imaging. Larger patient numbers are needed to truly evaluate the impact of surgical time on soft tissue changes and CK measurements. Other inflammatory markers such as interleukin-6 (IL-6) and C-reactive protein (CRP) have been used to assess post-operative tissue trauma in humans. While it has been shown that CRP and IL-6 increase after lumbar spine fixation in humans, other factors may contribute to an increase of these levels including age, stress, chronic conditions, blood loss during surgery, and visceral fat (42). However, these markers in addition to creatine kinase and post-operative MRI changes may have the potential to better assess differences in soft tissue injury between the two approaches.

The pain scores were widely variable between the two groups, but potential trends were noted. In the first 24 h post-operatively, group 1 showed a spike in pain scores on both the NPS and CMPS scales, which were still considered mild pain scores. After the first 48 h, the pain scores were similar among the two groups. Importantly, the CMPS considers behaviors that may not be pain-related, such as whining and restlessness. Overall, the MIS group had longer surgical times compared to the standard group. The impact surgical time and prolonged muscle retraction had on soft tissue injury and pain scores remains unclear. Other factors that could contribute to discrepancies in pain scores are patient behaviors (i.e., anxiety, aggression, fear) and side effects of opioid medication (i.e., dysphoria). Additionally, some dogs underwent a second anesthesia and received additional analgesia. Unless pain scales to account for patient behaviors are developed, differentiating true hyperesthesia will continue to be difficult for our veterinary patients. Measuring pain through more objective measures such as mechanical sensory threshold via algometry, would provide valuable information in future studies.

Drawing definitive conclusions about clinical outcome is beyond the scope of the present study. None of the patients in the MIS group were nociception negative at presentation nor showed neurologic worsening post-operatively. Whereas two patients from the standard open approach group lacked nociception at presentation and a third patient lost nociception post-operatively. In this study, 66.7% of patients that were nociception positive on presentation recovered ambulation compared with 50% of the patients that were nociception negative recovering ambulation, which is lower than other recovery rates based on nociception status. In the ACVIM consensus statement on diagnosis and management of acute canine thoracolumbar intervertebral disc extrusion, a review of surgical outcomes in dogs revealed a 93% recovery rate of deep pain positive dogs compared with a 61% recovery rate in deep pain negative dogs (43). Further studies are warranted to evaluate the outcomes in a large population of dogs for both approaches with equal numbers of varying neurologic grades and longer follow-up times.

Limitations of this study included the small study population, variability in timing of the anesthetized procedures and the analgesics used for pre-medication, using subjective measures of pain, inability to determine the degree of post-operative hemorrhage on MR imaging, and the inherent learning curve associated with minimally invasive surgery. All the patients in this study presented on an emergency basis to the CSU-VTH which means there was no control over the time of presentation. The availability of technical support staff to have a pre- and post-operative MRI performed, availability of board-certified radiologists, coordination with the anesthesia service, surgical start time and availability of endoscopic equipment all influenced the timeline of each patient. In most instances, a pre-operative MRI, surgery, and post-operative MRI were not all performed on the same day. The post-operative MRI was performed within 24–48 h of surgery. The length of time between surgery and post-operative imaging may have influenced the soft tissue changes evaluated on MRI as previously stated. Post-operative pain medication options were provided to limit variability across the two groups. This did not account for those patients that received a post-operative MRI on a different day than surgery as peri-operative analgesia is a normal component of the anesthesia protocol and withholding these medications would be unethical. Therefore, some patients received additional pain medication compared to those that had a post-operative MRI immediately after surgery. Additionally, pain was not measured through more objective means, e.g., mechanical sensory threshold via algometry, largely because pain evaluation was not a primary objective of the current study. As was discussed previously, there is a steep learning curve for minimally invasive procedures and this can directly affect surgical times, complication rates, and clinical outcomes. However, a larger study population, adhering to a standardized pain management protocol and improving surgical efficiency by continued cases, may all help further evaluate the previously proposed advantages of the MIS approach to the vertebral canal.

While the MIS approach was comparable to the open standard approach for this patient population, it is vital to understand its limitations and when it is most appropriate to perform. Factors that may limit the use of the MIS approach for a particular patient include the presence of multiple intervertebral disc extrusions, a widely dispersed single intervertebral disc extrusion, a chronic intervertebral disc protrusion, or involvement of the nerve roots. Other factors to consider are the equipment needed and the significant learning curve of this approach. While a surgeon is becoming familiar with the equipment, prolonged anesthesia and surgical times can be expected. This could further reduce case selection as it may be desirable to limit the anesthesia length for patients with underlying comorbidities. The steep learning curve of minimally invasive spinal surgery may be mitigated by having a high level of experience with the open approach and anatomy first, then by practicing on cadavers under the supervision of an experienced surgeon to become proficient with the equipment, work through a narrow surgical window, and become familiar with three-dimensional anatomy using an endoscope that projects a two-dimensional image. Additionally, training drills involving the entire surgical team, or a single operating room technician would help set-up of the equipment run more seamlessly.

## 5 Conclusion

Results of this study demonstrated that hemilaminectomies can be successfully completed using this MIS technique in dogs with naturally occurring intervertebral disc herniation. Additionally, the vertebral canal is readily accessible, and the surgeon can adequately remove extruded disc material and decompress the nervous tissue. Longer surgery times in the MIS group may be related to additional set-up requirements and the natural learning curve of the procedure. This may also have an impact on the soft tissue changes on post-operative neuroimaging, serum CK levels, and pain scores. Larger study populations are necessary to evaluate these factors, including postoperative pain.

## Data availability statement

The original contributions presented in the study are included in the article/Supplementary material, further inquiries can be directed to the corresponding author.

## Ethics statement

The animal studies were approved by Institutional Animal Care and Use Committee. The studies were conducted in accordance with the local legislation and institutional requirements. Written informed consent was obtained from the owners for the participation of their animals in this study.

## Author contributions

BM: Writing – original draft. LB: Conceptualization, Investigation, Methodology, Supervision, Visualization, Writing – review & editing. AM: Writing – review & editing. SR: Formal analysis, Writing – review & editing. ED: Writing – review & editing. TA: Writing – review & editing. EM: Conceptualization, Investigation, Writing – review & editing.
