# Supplementary material for: The use of a minimally invasive integrated endoscopic system to perform hemilaminectomies in chondrodystrophic dogs with thoracolumbar intervertebral disc extrusions
Source: Front Vet Sci. 2024 Apr 24;11:1296051. doi: 10.3389/fvets.2024.1296051 (PMC11078256; doi:10.3389/fvets.2024.1296051)
Supplement: Supplementary file 1 [file Data_Sheet_1.DOCX]

**Quality of Life Questionnaire**

Please complete a quality of life questionnaire once a day for the first 7 days. Return your completed copies to CSU’s Neurology Clinical Trials service at your 2-week recheck.

**Patient name: ______________ Date: _________ Your Name:___________________________**

| Rate 1-5 (1 = worst/low/not very, 5 = best/high/very) |
| --- |
| 1) How do you think your pet would rate its quality of life? |
| 1 2 3 4 5 |
| 2) To what extent do you feel that physical pain prevents your pet from doing what they need to do? |
| 1 2 3 4 5 |
| 3) What level of medical treatment does your dog require? |
| 1 2 3 4 5 |
| 4) Does your dog sleep through the night/have normal sleeping habits? |
| 1 2 3 4 5 |
| 5) How well is your pet able to get around? |
| 1 2 3 4 5 |
| 6) How much does your dog whine or cry? |
| 1 2 3 4 5 |
| 7) How difficult is it to limit your pet’s activity? |
| 1 2 3 4 5 |
| 8) My pet chews or scratches areas until they are red or irritated |
| 1 2 3 4 5 |
| 9) I am happy with my pet’s progress post operatively |
| 1 2 3 4 5 |
| 10) How painful do you feel your pet is? |
| 1 2 3 4 5 |

**Yes/No:**

| If instructed to manually express your dog’s bladder: |
| --- |
| 1) My pet is able to urinate easily with manual expression |
| YES NO |
| 2) My pet is able to urinate easily without manual expression |
| YES NO |

| Standing and walking: |
| --- |
| 3) My pet is able to stand |
| YES NO |
| 4) My pet is only able to stand when helped to a standing position |
| YES NO |
| 5) My pet is able to walk without support (able to take more than one step) |
| YES NO |
| 6) My pet is able to walk with support |
| YES NO |

| Pain: |
| --- |
| 7) My pet seems more uncomfortable than not |
| YES NO |
| 8) My pet is only painful when performing certain tasks |
| YES NO |

| Other (Only need to answer the first time completing this survey): |
| --- |
| 9) Have you ever had any experience dealing with other animals with a spinal cord injury? |
| YES NO |

**Additional Comments:**

____________________________________________________________________________________________________________________________________________________________________________________________________________________________________________________________________________________________________________________________________________________________________________________________________________________________________________________________________________________________________________________________
